# Potentially inappropriate drug use in myasthenia gravis: a real-world population-based cohort study in Italy

**DOI:** 10.3389/fneur.2023.1293626

**Published:** 2023-12-21

**Authors:** Giada Crescioli, Marco Finocchietti, Olga Paoletti, Paola Brunori, Francesco Sciancalepore, Marco Tuccori, Antonio Addis, Alfredo Vannacci, Niccolò Lombardi, Ursula Kirchmayer, The CAESAR study group

**Affiliations:** ^1^Department of Neurosciences, Psychology, Drug Research and Child Health, Section of Pharmacology and Toxicology, University of Florence, Florence, Italy; ^2^Tuscan Regional Center of Pharmacovigilance, Florence, Italy; ^3^Department of Epidemiology, Lazio Regional Health Service, Rome, Italy; ^4^Pharmacoepidemiology Unit, Regional Health Agency of Tuscany, Florence, Italy; ^5^Unit of Neurophysiopathology, Perugia Hospital, Perugia, Italy; ^6^National Center for Disease Prevention and Health Promotion, Italian National Institute of Health, Rome, Italy; ^7^Department of Clinical and Experimental Medicine, Unit of Pharmacology and Pharmacovigilance, University of Pisa, Pisa, Italy

**Keywords:** cohort study, contraindication, drug–drug interaction, drug use, myasthenia gravis, pharmacoepidemiology

## Abstract

**Introduction:**

To evaluate the use of pyridostigmine in presence of contraindications, and the use of concomitant potentially contraindicated drugs in a cohort of patients affected by Myasthenia Gravis (MG) in the Italian Regions of Lazio, Tuscany, and Umbria.

**Methods:**

This is a retrospective cohort study. A multivariate logistic regression model was used to evaluate the determinants of pyridostigmine and of potentially contraindicated drugs use in MG patients.

**Results:**

Among 591 incident pyridostigmine users affected by MG, 91 (15.4%) had at least one of the contraindications considered at the first prescription of pyridostigmine. Patients prescribed with pyridostigmine in presence of contraindications were more frequently affected by diabetes, obesity, and renal diseases. Age 75+ years (odds ratio, OR 4.94, 95% confidence interval, CI 1.60–15.22 for Latium; OR 3.78, 95%CI: 1.26–11.34 for Tuscany; OR 5.83, 95%CI 1.19–28.52 for Umbria), the presence of at least one specific comorbidity (OR 3.93; 95%CI 1.68–9.17 for Latium), and polytherapy (6+ drugs, OR 4.90, 95%CI: 1.35–17.85 for Tuscany) were found to be significantly associated with pyridostigmine use in presence of contraindications. Among patients affected by MG, 1,483 (62.6%) were treated with potentially contraindicated drugs in the first year of follow-up (67.06.9% in Latium; 59% in Tuscany; 57.6% in Umbria). Patients aged 75+ years, those with at least one specific complication or comorbidity, and those exposed to polytherapy were more likely to be treated with a potential contraindicated drug.

**Conclusion:**

Among incident users of pyridostigmine, more than 15% of patients have at least one of the contraindications considered, and among patients diagnosed with MG, in the first year of follow-up >60% of subjects were treated with potentially contraindicated drugs.

## 1 Introduction

Myasthenia gravis (MG) is a chronic autoimmune neuromuscular disease that affects the transmission of signals between nerve and muscle cells (1). MG causes weakness and fatigue of various voluntary muscles, such as those controlling eye movements, facial expressions, chewing, swallowing, speaking, and breathing (2). The symptoms of MG may vary in type and severity depending on the muscles involved and the level of autoantibody production. MG can be classified into different subtypes based on the age of onset, the presence or absence of a thymic tumor (thymoma), and the type of autoantibody detected in the blood (3). The most common subtype is autoimmune MG with antibodies against acetylcholine receptors (AChR), which accounts for about 80% of cases. Other subtypes include MG with antibodies against muscle-specific kinase (MuSK), low-density lipoprotein receptor-related protein 4 (Lrp4), or agrin; MG with no detectable antibodies (seronegative MG); and congenital or genetic MG (4). The diagnosis of MG is based on clinical features, neurophysiological tests, serological tests, and imaging studies. The treatment of MG aims to improve muscle strength and function, reduce autoantibody production, and remove or suppress the abnormal thymus gland (5). The main therapeutic options include anticholinesterase drugs, immunosuppressive agents, intravenous immunoglobulin (IVIg), plasma exchange (PLEX), and thymectomy.

In clinical practice, several drugs are known to be potentially inappropriate for MG. A number of medications such as immune checkpoint inhibitors, penicillamine, tyrosine kinase inhibitors, and interferons may induce *de novo* MG by altering the immune homeostasis mechanisms which prevent emergence of autoimmune diseases such as MG (6). Specifically, these medications can trigger autoimmunity, leading to symptomatic MG. Other drugs, for example certain antibiotics (i.e., ketolides, fluoroquinolones, macrolides, and aminoglycosides), cardiovascular drugs (i.e., beta-blocking agents, calcium channel blockers, and antiarrhythmics), anesthetics and neuromuscular blockers, have deleterious effects on neuromuscular transmission and have been linked to the deterioration of MG symptoms, potentially causing MG crises or revealing previously undiagnosed cases (6). For some of these drugs, especially for fluoroquinolones (i.e., ciprofloxacin, moxifloxacin, and levofloxacin), the United States Food and Drug Administration (FDA) has issued a black box warning for their use in MG (7), reporting that these treatments should not be used in this condition, or they should be used cautiously if no alternative treatment is available. Patients should be monitored carefully for this possibility and dose adjustments may be needed (8). Patients with MG should be aware of the potential risks of the aforementioned drugs and avoid them if possible or use them with caution and under strict medical supervision.

Considering drugs indicated for MG, first line therapy is represented by anticholinesterase medications, in particular pyridostigmine (ATC code N07AA02). By increasing the amount of acetylcholine available, pyridostigmine can improve the function of muscles that are affected by neuromuscular disorders such as MG (9). However, pyridostigmine is not without contraindications and potential side effects. Pyridostigmine is contraindicated in patients with known hypersensitivity to anticholinesterase agents, and mechanical intestinal or urinary obstruction (10). Pyridostigmine can also interact with other medications that affect cholinergic or neuromuscular transmission, such as beta-blockers, calcium channel blockers, anticholinergics, opioids, aminoglycosides, and quinolones (6). Therefore, pyridostigmine should be used with caution in patients with these conditions or medications, requiring careful dosing and monitoring to avoid complications and potential drug–drug interactions (DDIs) (11).

In light of this, and considering that the real-world evidence regarding the use of contraindicated medications in MG are still lacking, the aim of the present study was to describe the use potentially inappropriate medications, both among those indicated and contraindicated, in a cohort of Italian subjects diagnosed with MG.

## 2 Methods

### 2.1 Study design

This is a real-world population-based retrospective cohort study, part of a multiregional Italian pharmacovigilance project on the comparative effectiveness and safety of drugs used in rare neuromuscular and neurodegenerative diseases (the CAESAR project) (12). In the period 2013–2019, three Italian regions were involved: Latium, Tuscany, and Umbria. The study protocol was registered on the EU PAS Register (EUPAS37983).

### 2.2 Data sources

In Italy, healthcare is funded through taxes and extends services to all residents registered in healthcare registries, encompassing approximately 95% of the population. Non-enrolment primarily pertains to healthy individuals in their youth or middle age who can afford private medical care, while those with chronic conditions are generally covered. For this demographic, all healthcare services offered by public or affiliated providers are documented at an individual level. Analyses were performed on regional administrative healthcare databases: (1) the healthcare assistance registry, containing demographic and residence information of subjects assisted by the regional healthcare system; (2) the hospital discharge database, which includes information on hospital admissions and discharges, with principal and secondary diagnosis and procedures, coded by ICD-9-CM classification; (3) emergency department (ED) visits (data relating to ED visits, including principal and secondary diagnoses, coded according to the ICD-9-CM classification, patients conditions, and triage parameters); (4) drug dispensing registry, containing information on drugs reimbursed by the healthcare system, including dispensation date and active substance, coded according to ATC classification; (5) co-payment exemptions for subjects with a diagnosis of a chronic or acute condition, including start and end dates of the exemptions; and (6) mortality information system, which includes information of date and place of death. All databases can be linked at regional level by considering an anonymous subject identifier.

### 2.3 Cohort selection

We considered all subjects with these inclusion criteria: (a) discharge from hospital with a primary diagnosis of MG (ICD-9-CM code 358.0) or a secondary diagnosis in combination with discharge from a hospital neurology ward; (b) discharge from ED with a primary diagnosis of MG (ICD-9-CM code 358.0); and (c) disease specific co-payment exemption for MG (Italian code: RFG101).

#### 2.3.1 Pyridostigmine in presence of potential contraindications

The aim of the first part of the study was to evaluate eventual determinants of pyridostigmine use in presence of contraindications (defined as potentially inappropriate use). Starting from the cohort of MG subjects in the period 2013–2019, we only included 18 years of age or older, resident in the three Italian regions, present in the healthcare assistance database at the index date (date of MG diagnosis) and in the 2 years before (look-back period). Then we selected incident users of pyridostigmine after the index date. Incident users were defined as subjects with at least one dispensation of pyridostigmine after the index date and no dispensations in the 2 years before. Incident users were divided in two different cohorts: (1) subjects starting pyridostigmine treatment in presence of contraindications, namely mechanical gastrointestinal or mechanical urinary obstruction, or obstructive respiratory diseases, or cardiovascular diseases, or care involving breathing exercises, retrieved in the 2 years before the first dispensation of pyridostigmine (details in Supplementary Table 1); (2) subjects starting pyridostigmine treatment without any trace of these contraindications. Selection criteria and final cohorts are shown in flowcharts. These two cohorts were characterized and compared in terms of demographic characteristics (sex and age), comorbidities, complications, pharmacological and non-pharmacological therapies, applying univariate frequency distributions represented in tables.

#### 2.3.2 Potentially contraindicated concomitant medications

The second part of the study focused on the use of drugs with potential contraindications for subjects diagnosed with MG (defined as potentially inappropriate use). From the total cohort of MG subjects in the period 2013–2019, 18 years of age or older, resident and present in healthcare assistance database at the index date and in the 2 years before were enrolled. Successively, cohort was divided into: (1) subjects diagnosed with MG and using at least one potentially contraindicated drug (i.e., drugs for the cardiovascular system, such as beta blocking agents, calcium channel blockers, procainamide, disopyramide, statins; drugs for the nervous system, such as antiepileptics, antipsychotics, antidepressants; imm and unosuppressants; details in Supplementary Table 2) in the first year after the index date; (2) subjects diagnosed with MG and without dispensations of potential contraindicated drugs in the first year after the index date. Criteria for selecting the two cohorts as well as the total number of the two groups are represented in flowcharts. The abovementioned two cohorts were characterized in terms of demographic characteristics (sex and age) and clinical characteristics (i.e., comorbidities, complications, pharmacological and non-pharmacological therapies), by using univariate frequency distributions. Results are shown in tables.

### 2.4 Statistical analysis

Separately for each of the two parts, the cohorts were compared through univariate frequency distributions. For each variable, the Pearson’s chi-squared test was computed to assess differences between the two cohorts. A univariate logistic regression analysis was computed to identify determinants of potentially inappropriate therapy for both, use of pyridostigmine in presence of potential contraindications, and use of potentially contraindicated drugs in patients with MG, separately for each region. Results are shown in forest plots. All ICD-9-CM codes used are available in Supplementary Table 3.

## 3 Results

### 3.1 Use of pyridostigmine in presence of potential contraindications

**Figure 1:**
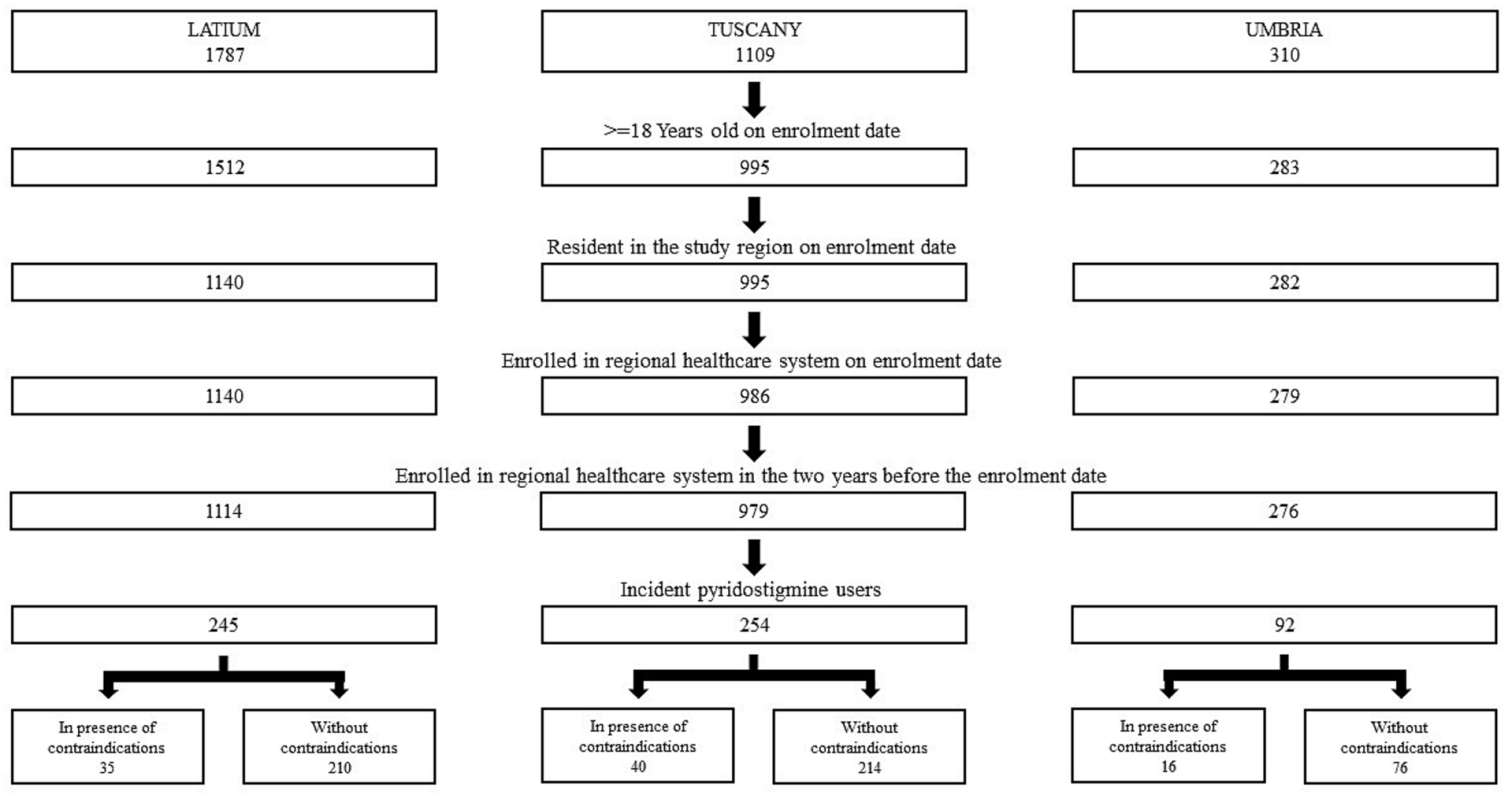
Cohort selection of incident users of pyridostigmine in three Italian regions in the period 2013–2019.

After applying inclusion and exclusion criteria, a total of 591 incident users of pyridostigmine were identified (Figure 1). For 91 of these, at least one of the considered contraindications were present at first pyridostigmine prescription.

Patient characteristics are summarized in Table 1. Both cohorts comprised more males (58.2% in the cohort of incident users in the presence of potential contraindications, 56.6% in the one without contraindications), and the majority was aged above 65 years (83.5 and 56.6% respectively).

**Table 1:** Baseline characteristics of incident users of pyridostigmine in presence or absence of contraindications.

	Presence of contraindications		No contraindications		*p* value
	91		500		
	*n*	%	*n*	%	
Demographical characteristics					
Sex					
Males	53	58.2%	283	56.6%	0.771
Females	38	41.8%	217	43.4%	
Age					
18–64	15	16.5%	217	43.4%	<0.001^*^
65–74	25	27.5%	134	26.8%	
75+	51	56.0%	149	29.8%	
Specific complications					
Acute respiratory failure	15	16.5%	15	3.0%	<0.001^*^
Myasthenia gravis with (acute) exacerbation	53	58.2%	281	56.2%	0.717
Benign neoplasm of thymus	3	3.3%	13	2.6%	0.706
Malignant neoplasm of thymus	7	7.7%	10	2.0%	0.003^*^
Any specific complication	61	67.0%	291	58.2%	0.114
Specific comorbidities					
Thyrotoxicosis	2	2.2%	0	0.0%	<0.001^*^
Diabetes mellitus without mention of complication	20	22.0%	29	5.8%	<0.001^*^
Fractures	7	7.7%	28	5.6%	0.437
Other and unspecified non-infectious gastroenteritis and colitis	2	2.2%	2	0.4%	0.054
Other and unspecified hyperlipidemia	2	2.2%	3	0.6%	0.126
Overweight, obesity and other hyperalimentation	6	6.6%	6	1.2%	<0.001^*^
Autoimmune diseases	1	1.1%	15	3.0%	0.314
Any specific comorbidity	34	37.4%	96	19.2%	<0.001^*^
Other comorbidities					
Liver diseases	2	2.2%	3	0.6%	0.126
Renal diseases	5	5.5%	6	1.2%	0.005^*^
Neoplasms	18	19.8%	55	11.0%	0.019^*^
Mental disorders	11	12.1%	33	6.6%	0.067
Diseases of the circulatory system	37	40.7%	185	37.0%	0.507
Diseases of the respiratory system	25	27.5%	48	9.6%	<0.001^*^
Any other comorbidity	59	64.8%	252	50.4%	0.011^*^
Pharmacological therapy					
Prednisone (ATC code: H02AB07)	64	70.3%	309	61.8%	0.121
Vitamin D (ATC code: A11CC)	20	22.0%	128	25.6%	0.463
Azathioprine (ATC code: L04AX01)	4	4.4%	44	8.8%	0.157
At least one specific drug	68	74.7%	362	72.4%	0.647
Combinations of specific drugs					
Azathioprine	0	0.0%	8	1.6%	0.362
Azathioprine, Prednisone	4	4.4%	21	4.2%	
*Azathioprine, Prednisone, Vitamin D*	0	0.0%	13	2.6%	
*Azathioprine, Vitamin D*	0	0.0%	2	0.4%	
*Prednisone*	44	48.4%	202	40.4%	
*Prednisone, Vitamin D*	16	17.6%	73	14.6%	
*Vitamin D*	4	4.4%	43	8.6%	
*None of these therapies*	23	25.3%	138	27.6%	
Other omedications (ATC 4th level)					
*0–5*	14	15.4%	176	35.2%	<0.001^*^
*6+*	77	84.6%	324	64.8%	
Non-pharmacological therapy					
Thymectomy	3	3.3%	7	1.4%	0.197
Non-invasive mechanical ventilation	2	2.2%	2	0.4%	0.054
Invasive mechanical ventilation	12	13.2%	13	2.6%	<0.001^*^
Mechanical ventilation	14	15.4%	14	2.8%	<0.001^*^
Plasmapheresis	4	4.4%	36	7.2%	0.327
Any non-pharmacological therapy	20	22.0%	49	9.8%	<0.001^*^

^*^Specific comorbidities with frequencies <1% are not displayed (chronic lymphocytic thyroiditis, systemic lupus erythematosus, rheumatoid arthritis, pernicious anemia, other chronic hepatitis, psoriasis and similar disorders, diabetes with neurological manifestations, malignant essential hypertension, toxic cataract, other osteoporosis, Cushing’s syndrome, poisoning with parasympathomimetics, obstructive chronic bronchitis, other specified cardiac dysrhythmias, cramp of limb). ^**^Other comorbidities with frequencies <1% are not displayed (inflammatory diseases of the central nervous system).

Myasthenia Gravis specific complications were retrieved in both cohorts, with higher frequencies in patients with potential contraindications (one or more contraindications 67.0 vs. 58.2%). In particular, a significant difference emerged for acute respiratory failure (16.5 and 3.0% respectively) and malignant neoplasm of thymus (7.7 and 2.0% respectively).

Differences between cohorts were also pronounced regarding the presence of specific comorbidities (37.4 vs. 19.2%). In particular, significantly higher proportions in patients with contraindications for diabetes mellitus without mention of complication (22.0 and 5.8%) and overweight, obesity, and other hyperalimentation (6.6 vs. 1.2%) were observed.

A significant statistical difference in the presence of other comorbidities was also observed (64.8 vs. 50.4%), in particular diseases of the respiratory system (27.5 vs. 9.6%), neoplasms (19.8 vs. 11.0%), and renal diseases (5.5 vs. 1.2%).

Regarding specific drug therapy, pyridostigmine was used in monotherapy in about one quarter of the patients in both cohorts (25.3 and 27.6%). The majority of patients received prednisone (70.3 and 61.8%) or vitamin D (22.0 and 25.6%), while azathioprine was scarcely used (4.4 and 8.8%). Combinations mainly yielded at the use of pyridostigmine with prednisone alone (48.4 and 40.4%), or with prednisone and vitamin D (17.6 and 14.6%). Among other comedications, polytherapy (6+ drugs) was more frequent in patients with contraindications (84.6 vs. 64.8%).

Also, non-pharmacological therapies were more frequent in presence of contraindications (22.0 vs. 9.8%), in particular mechanical ventilation (15.4 vs. 2.8%) and invasive mechanical ventilation (13.2 vs. 2.6%).

**Figure 2:**
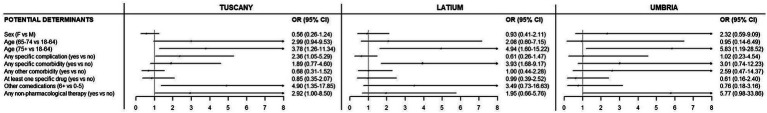
Determinants of receiving pyridostigmine in the presence of contraindications.

The analysis of determinants of receiving pyridostigmine in the presence of contraindications gave evidence for indicators of major fragility (Figure 2), such as increasing age (75 or higher vs. 18–64 years old) in Latium [OR = 4.94, 95%CI: 1.60–15.22], in Tuscany [OR = 3.78, 95%CI: 1.26–11.34] and in Umbria [OR = 5.83, 95%CI: 1.19–28.52], the presence of at least one specific complication in Tuscany [OR = 2.36, 95%CI: 1.05–5.29], the presence of at least one specific comorbidity in Latium [OR = 3.93, 95%CI: 1.68–9.17], polytherapy of other comedications (6+ vs. 0–5) [OR = 4.90, 95%CI: 1.35–17.85], and the presence of non-pharmacological therapy [OR = 2.92, 95%CI: 1.00–8.50] in Tuscany.

### 3.2 Use of potentially contraindicated concomitant medications

**Figure 3:**
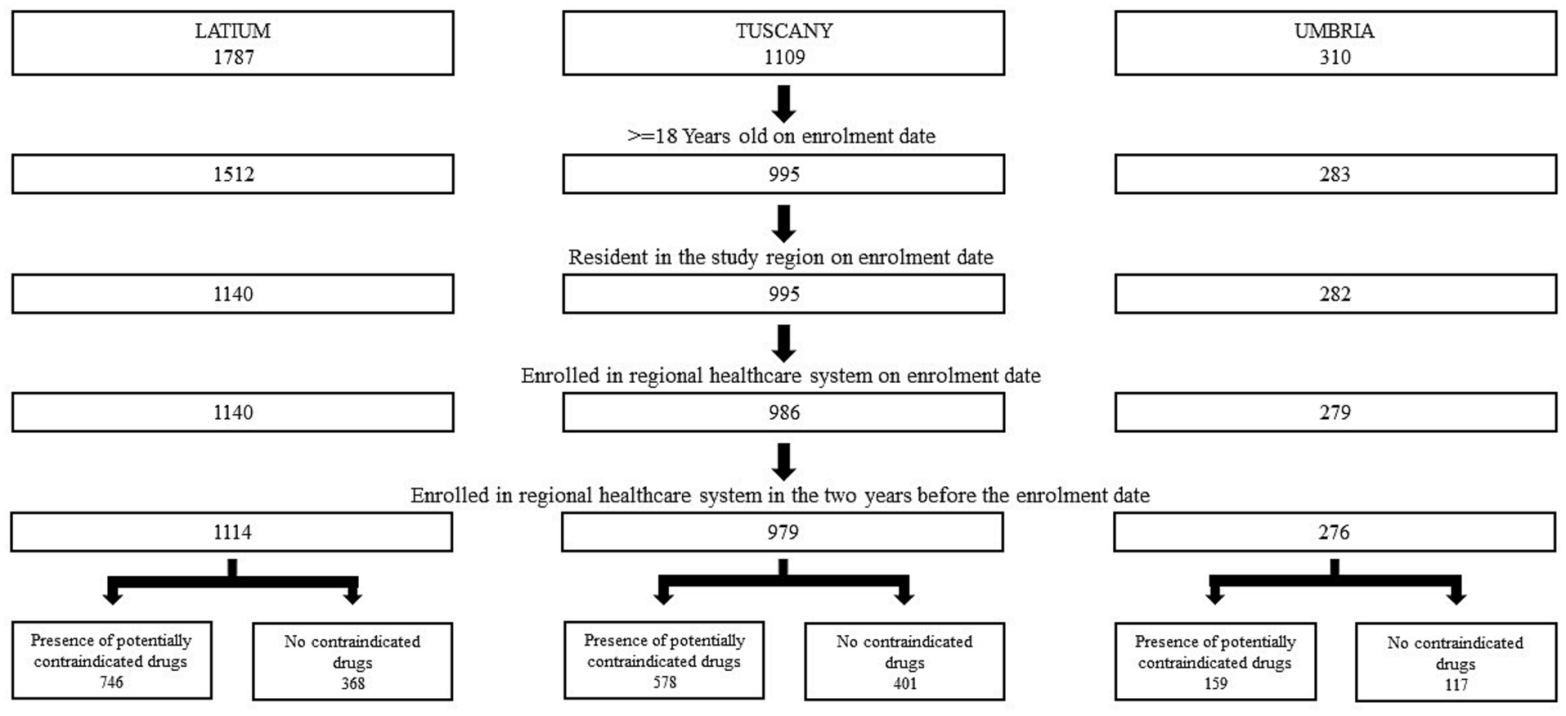
Cohort selection of MG patients in three Italian regions in the period 2013–2019.

A total number of 2,369 MG subjects was enrolled, of whom 1,483 were prescribed with potentially contraindicated drugs in the first year (Figure 3).

Patient characteristics are summarized in Table 2. Compared to the 886 subjects without potentially contraindicated drugs, these patients were older (62.6 vs. 38.2% were 65 or more years old respectively), and more often affected by specific complications (46.2% had at least one complication compared to the 35.2% of the other cohort). In particular, myasthenia gravis with (acute) exacerbation (43.4 vs. 33.1%) and acute respiratory failure (5.3 vs. 2.9%). Cohort prescribed with potentially contraindicated drugs suffered more of specific comorbidities (22.5 vs. 12.6%), in particular diabetes mellitus without mention of complication (7.7 vs. 3.3%), obstructive chronic bronchitis (4.5 vs. 1.2%). A significant statistical difference in the presence of at least one other comorbidity was also observed (48.1 vs. 27.7%), in particular diseases of the circulatory system (35.9 vs. 17.6%), diseases of the respiratory system (13.1 vs. 8.0%), and neoplasms (10.7 vs. 6.4%).

**Table 2:** Baseline characteristics of MG patients with or without prescriptions of potentially contraindicated drugs.

	Subjects without potentially contraindicated drugs		Subjects with potentially contraindicated drugs		*p* value
	886		1,483		
	*n*	%	*n*	%	
Demographical characteristics					
Sex					
Males	420	47.4%	744	50.2%	0.193
Females	466	52.6%	739	49.8%	
Age					
18–64	548	61.9%	555	37.4%	<0.001^*^
65-74	170	19.2%	423	28.5%	
75+	168	19.0%	505	34.1%	
Specific complications					
Acute respiratory failure	26	2.9%	79	5.3%	0.006^*^
Myasthenia gravis with (acute) exacerbation	293	33.1%	643	43.4%	<0.001^*^
Malignant neoplasm of thymus	13	1.5%	34	2.3%	0.163
Any specific complication	312	35.2%	685	46.2%	<0.001^*^
Specific comorbidities					
Diabetes mellitus without mention of complication	29	3.3%	114	7.7%	<0.001^*^
Fractures	37	4.2%	103	6.9%	0.006^*^
Obstructive chronic bronchitis	11	1.2%	67	4.5%	<0.001^*^
Overweight, obesity and other hyperalimentation	10	1.1%	30	2.0%	0.102
Autoimmune diseases	18	2.0%	32	2.2%	0.836
Any specific comorbidity	112	12.6%	333	22.5%	<0.001^*^
Other comorbidities					
Renal diseases	9	1.0%	49	3.3%	<0.001^*^
Neoplasms	57	6.4%	159	10.7%	<0.001^*^
Mental disorders	36	4.1%	113	7.6%	<0.001^*^
Diseases of the circulatory system	156	17.6%	532	35.9%	<0.001^*^
Diseases of the respiratory system	71	8.0%	194	13.1%	<0.001^*^
Any other comorbidity	245	27.7%	714	48.1%	<0.001^*^
Pharmacological therapy					
Pyridostigmine (ATC code: N07AA02)	390	44.0%	802	54.1%	<0.001^*^
Prednisone (ATC code: H02AB07)	348	39.3%	791	53.3%	<0.001^*^
Vitamin D (ATC code: A11CC)	266	30.0%	583	39.3%	<0.001^*^
Azathioprine (ATC code: L04AX01)	13	1.5%	203	13.7%	<0.001^*^
At least one specific drug	558	63.0%	1,134	76.5%	<0.001^*^
Combinations of specific drugs					
*Azathioprine*	0	0.0%	6	0.4%	-
*Azathioprine, Prednisone*	0	0.0%	6	0.4%	
*Azathioprine, Prednisone, Pyridostigmine*	0	0.0%	52	3.5%	
*Azathioprine, Prednisone, Pyridostigmine, Vitamin D*	5	0.6%	95	6.4%	
*Azathioprine, Prednisone, Vitamin D*	3	0.3%	13	0.9%	
*Azathioprine, Pyridostigmine*	3	0.3%	14	0.9%	
*Azathioprine, Pyridostigmine, Vitamin D*	2	0.2%	13	0.9%	
*Azathioprine, Vitamin D*	32	3.6%	4	0.3%	
*Prednisone*	55	6.2%	104	7.0%	
*Prednisone, Pyridostigmine*	112	12.6%	229	15.4%	
*Prednisone, Pyridostigmine, Vitamin D*	114	12.9%	208	14.0%	
*Prednisone, Vitamin D*	59	6.7%	84	5.7%	
*Pyridostigmine*	122	13.8%	140	9.4%	
*Pyridostigmine, Vitamin D*	33	3.7%	51	3.4%		
*Vitamin D*	50	5.6%	115	7.8%	
*None of these therapies*	328	37.0%	349	23.5%	
Other omedications (ATC 4th level)					
*0–5*	517	58.4%	359	24.2%	<0.001^*^
*6+*	369	41.6%	1,124	75.8%	
Non-pharmacological therapy					
Invasive mechanical ventilation	15	1.7%	42	2.8%	0.080
Mechanical ventilation	15	1.7%	49	3.3%	0.019^*^
Plasmapheresis	11	1.2%	64	4.3%	<0.001^*^
Any non-pharmacological therapy	34	3.8%	127	8.6%	<0.001^*^

^*^Specific complications with frequencies <1% are not displayed (benign neoplasm of thymus). ^**^Specific comorbidities with frequencies <1% are not displayed (thyrotoxicosis, chronic lymphocytic thyroiditis, systemic lupus erythematosus, rheumatoid arthritis, pernicious anemia, chronic and recurrent hepatitis, psoriasis and similar disorders, diabetes with neurological manifestations, malignant essential hypertension, toxic cataract, other osteoporosis, Cushing’s syndrome, poisoning with parasympathomimetics, other and unspecified non-infectious gastroenteritis and colitis, other specified cardiac dysrhythmias, other and unspecified hyperlipidaemia, cramp of limb). ^***^Other comorbidities with frequencies <1% are not displayed (liver diseases, inflammatory diseases of the central nervous system).

Patients with potentially contraindicated drugs were also more frequently treated with MG drug therapy, i.e., pyridostigmine (54.1 and 44.0%), prednisone (53.3 and 39.3%), vitamin D (39.3 and 30.0%), and azathioprine (13.7 and 1.5%). Similar patterns were observed for most of the combined therapies (76.5 vs. 63.0%) and for polytherapy (6+ drugs) of other comedications (75.8 vs. 41.6%).

A statistical difference in presence of at least one of the non-pharmacological therapies was also observed (8.6 vs. 3.8%).

**Figure 4:**
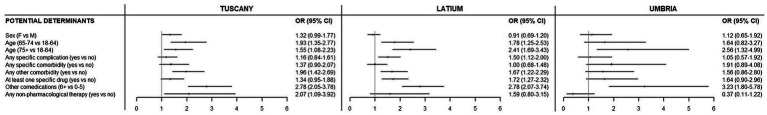
Determinants for the use of potentially contraindicated drugs.

Results of the analysis of eventual determinants for the use of contraindicated drugs in the presence of myasthenia gravis for each region are shown in Figure 4. For Latium, increasing age [OR = 1.78, 95%CI: 1.25–2.53 for the comparison 65–74 vs. 18–64, and OR = 2.41, 95%CI: 1.69–3.43 for the comparison 75+ vs. 18–64], presence of at least 1 specific complication [OR = 1.50, 95%CI: 1.12–2.00], presence of at least one other comorbidity [OR = 1.67, 95%CI: 1.22–2.29], use of specific pharmacological therapy [OR = 1.72, 95%CI: 1.27–2.32], and polytherapy (6+ vs. 0–5) of other comedications [OR = 2.78, 95%CI: 2.07–3.74] represented determinant factors for the use of potentially contraindicated drugs in presence of MG. As regard Tuscany, determinant covariates were: increasing age [OR = 1.93, 95%CI: 1.35–2.77 for the comparison 65–74 vs. 18–64, and OR = 1.55, 95%CI: 1.08–2.23 for the comparison 75+ vs. 18–64], presence of at least 1 other comorbidity [OR = 1.96, 95%CI: 1.42–2.69], polytherapy (6+ vs. 0–5) of other comedications [OR = 2.78, 95%CI: 2.05–3.78]. For Umbria, increasing age [OR = 2.56, 95%CI: 1.32–4.99 for the comparison 75+ vs. 18–64] and polytherapy of other comedications (6+ vs. 0–5) [OR = 3.23, 95%CI: 1.80–5.78] represented determinant factors for the use of potentially contraindicated drugs in presence of MG.

## 4 Discussion

The present study investigated use patterns of pyridostigmine in presence of potential contraindications and of potentially contraindicated concomitant medications in a real-world setting of MG. This pharmaco-epidemiological analysis was carried out in the three Italian regions of Lazio, Tuscany and Umbria participating in the CAESAR project, which used the administrative data of over 10 million residents. From a literature review, this is the first drug utilization study of its kind performed in Italy.

Pyridostigmine is the most commonly used medication in the symptomatic treatment of MG, however, research into its use in clinical practice and safety is still lacking. Using pyridostigmine in presence of contraindications may lead to treatment discontinuation, ineffectiveness of the therapy, and adverse drug reactions (ADRs). A cross-sectional study performed using the Dutch–Belgian myasthenia patient registry assessed the effectiveness, prevalence of side effects of pyridostigmine in 642 MG patients (9). Authors stated that 61% of patients reported that they currently used pyridostigmine, 36% had discontinued pyridostigmine, and 2% reported to never have used pyridostigmine. Of interest, 91% of them reported ADRs. Most frequently reported ADRs were gastrointestinal and urinary symptoms, followed by muscle cramps, blurred vision and hyperhidrosis. Among patients who discontinued pyridostigmine 26% reported that ADRs were the main reason for discontinuation. Even if this study did not report information regarding the use of pyridostigmine in presence of potential contraindications, considering that most of the ADRs were related to the gastrointestinal and urinary systems, it is likely that in this sample pyridostigmine was used in the presence of potential contraindications. Since our analysis focused on the presence of potential contraindications, while the study by Remijn-Nelissen and colleagues focused on the evaluation/description of ADRs, in the future it would be interesting to evaluate both the aforementioned safety aspects within the same cohort of patients.

Myasthenia Gravis individuals are usually prescribed with doses of 180 up to 480 mg daily of pyridostigmine. ADRs from pyridostigmine are frequently reported and usually dose dependent, and can necessitating a reduction of dose or slower titration (13). From a pharmacological point of view, the majority of pyridostigmine-related ADRs arise from its action on the muscarinic peripheral synapses, in particular those outside the neuromuscular junction, and symptoms are caused by an increase in cholinergic transmission (14). Taking into account the presence of contraindications, some patients can also be sensitive to the cardiac adverse events even with low doses of pyridostigmine, as well as some asthmatic patients who may experience bronchospasm during the therapy with this specific enzyme inhibitor (13). These occurrences are more frequently observed in frail subset, in fact, as we reported in our cohort, individuals prescribed with pyridostigmine in presence of contraindications were more likely to be affected by comorbidities (i.e., diabetes, obesity, and renal diseases), older (75+ years), and exposed to polytherapy (6+ drugs). Considering that pyridostigmine-related ADRs are common, the presence of contraindications when prescribing pyridostigmine may increase their occurrence. In light of this, when pyridostigmine administration is required, physicians should closely monitor their patients, especially those with contraindications, to maintain an adequate benefit/risk profile.

The use of specific medications and over-the-counter products, as well as complementary and alternative medicine (CAM) products (i.e., dietary supplements, phytotherapy products, etc.), can potentially exacerbate the symptoms of MG, and for this reason they are considered potentially contraindicated treatments for this clinical condition (15). Therefore, it is crucial to inform any healthcare professional, including the community pharmacist, about MG diagnosis, in particular prior to initiating any new medication/product.

As reported in the literature and in clinical guidelines (16) (17), several medications have been linked to the deterioration of MG symptoms. However, these associations should not automatically result in the exclusion of these medications for MG patients. In most cases, instances of MG worsening due to these drugs are exceedingly rare. Moreover, the evaluation of the causality assessment highlighted that the connection between the potentially contraindicated drugs/products and MG worsening may be coincidental rather than causal. Additionally, some medications may be essential for a MG patient’s treatment and should not be considered entirely prohibited. It is advisable for patients and physicians to acknowledge and discuss the potential of a particular drug exacerbating MG symptoms. They should also evaluate the advantages and disadvantages of alternative treatments, if available. If a new medication is initiated and the MG symptoms worsen, it is vital for the patient to promptly inform their physicians.

Among these groups of pharmacological treatments, the strongest evidence suggesting a connection with MG deterioration are available for drugs for the cardiovascular system (i.e., beta blocking agents, calcium channel blockers, some antiarrhythmics, statins, etc.), drug for the central nervous system (i.e., antiepileptics, antipsychotics, antidepressants, etc.), immunosuppressants (i.e., immune checkpoint inhibitors), and anti-infectives for systemic use (i.e., fluoroquinolones, macrolides, aminoglycosides, etc.).

Calcium channel blockers can potentially worsen MG symptoms due to their effects on the neuromuscular junction and muscle function (18). These medications inhibit the entry of calcium ions into cells by blocking calcium channels, which affects muscle contraction. In MG, where acetylcholine receptors are targeted by the immune system, calcium influx through these channels is crucial for acetylcholine release and normal neuromuscular transmission (6). By blocking calcium channels, these medications interfere with acetylcholine release, exacerbating impaired neuromuscular transmission in MG. Additionally, reduced muscle contraction occurs as calcium is essential for muscle function (19). Calcium channel blockers can also interact with other MG medications, potentially impacting their effectiveness or causing ADRs.

Another group of medications widely used in the general population is represented by beta-blockers. These medications block beta-adrenergic receptors, reducing the effects of adrenaline and causing physiological changes. In MG, where the immune system targets the neuromuscular junction, leading to muscle weakness, beta-blockers can interfere with the communication between nerves and muscles by blocking acetylcholine receptors (19). This disruption exacerbates MG symptoms. Additionally, beta-blockers can mask early signs of MG exacerbation by reducing heart rate and blood pressure, potentially delaying the detection of muscle weakness. It is important to note that the effects of beta blockers on MG vary, and the severity of the condition differs among individuals.

Statins, medications used to lower cholesterol levels, have been associated with rare cases of exacerbating MG symptoms. The exact mechanisms are not fully understood, but there are a couple of possible explanations (20). Statins have immune-modulating effects that may influence the immune response in MG, potentially worsening symptoms. Additionally, statins can cause muscle-related ADRs, such as pain, weakness, and muscle breakdown, which could further worsen MG symptoms. However, it is important to note that statin-related MG worsening is rare, and most individuals with MG can safely use statins without ADRs. Since statins are commonly prescribed for cardiovascular health, the decision to use them in individuals with MG should be based on a thorough discussion between the individual and their healthcare provider, considering the potential risks and benefits.

Procainamide is a drug that is used for irregular heart rhythm. It can impair neuromuscular transmission by blocking the postsynaptic acetylcholine receptors or inhibiting acetylcholine release, worsening MG and, for this reason, it should be used with caution (21).

Another important class of medication that should be used with caution in MG is represented by anti-infectives for systemic use. Aminoglycosides (i.e., gentamicin) and macrolides (i.e., erythromycin) can block the release of acetylcholine at the neuromuscular junction, leading to muscle weakness and exacerbating MG symptoms (22) (19). Fluoroquinolones (i.e., ciprofloxacin) have been reported to cause muscle weakness and MG exacerbations (23), possibly through their effects on acetylcholine receptors and neuromuscular transmission. Fluoroquinolones should be used cautiously, if at all, in MG, and the US FDA has issued a black box warning for their use in MG (24, 25). Tetracyclines (i.e., doxycycline) have been associated with a rare condition called “drug-induced myasthenia,” where muscle weakness similar to MG can develop. However, the exact mechanisms of tetracycline-induced myasthenia are not well understood (19). It’s important to note that not all antibiotics have the same effects on MG, and the likelihood of MG exacerbations from antibiotics is generally low. Most individuals with MG can safely use antibiotics, when necessary, but healthcare providers should be informed about the MG diagnosis before initiating antibiotic treatment. Close monitoring of MG symptoms and prompt reporting of any worsening to the healthcare provider are recommended during antibiotic therapy. In some cases, alternative antibiotics that are less likely to affect neuromuscular transmission may be considered (6).

Corticosteroids are commonly used to treat MG, but they can also cause transient worsening of the disease, with symptoms occurring over a few days to months (26). Among factors associated with MG worsening in patients treated with corticosteroids several studies found older age, predominant bulbar symptoms, presence of thymoma/thymectomy, dose of prednisone of more than 40 mg/day (27). The mechanism behind the transient worsening of MG with corticosteroids use is not fully understood, but researchers hypothesized that an initial increase in antibody production before immunosuppression takes effect could be the cause of MG worsening. When corticosteroids are introduced, they disrupt the balance between autoantibodies and regulatory mechanisms, temporarily increasing autoantibody activity. This exacerbation is usually temporary and improves with continued treatment. In these cases, patients should be monitored carefully and dose adjustments or tapering may be needed (28).

Among CAM products that could exacerbate MG symptoms, dietary supplements or foods containing high amounts of magnesium can cause severe muscle weakness, interfering with neuromuscular transmission by reducing calcium influx into the presynaptic terminals or increasing potassium efflux from the postsynaptic terminals (6) (29). Moreover, *Cinchona officinalis* and cinchona-based products (i.e., liqueurs) can cause respiratory depression in patients affected by MG, as well as quinine, an antimalarial drug that is sometimes used for leg cramps. Active principles derived from *Cinchona officinalis* can inhibit the neuromuscular transmission by blocking the postsynaptic acetylcholine receptors or preventing action potential transmission (30). Therefore, healthcare professionals, including the community pharmacist, should always be aware of the potential safety issues related to the use of CAM products in MG individuals.

### 4.1 Limitations and strengths

The primary limitation of our study arises from the inherent characteristics of data, which lack detailed clinical information like specific disease onset, duration, severity, and subtype of MG. It is important to note that the index date (date of diagnosis of MG) may not necessarily coincide with the actual initiation of the disease. These limitations can have different effects across the three Italian regions, as the traceability of drug claims depends on administrative procedures associated with drug registration under diverse reimbursement policies. Moreover, drugs given to hospitalized patients and those obtained without prescriptions (over-the-counter medications) generally cannot be traced at the individual patient level. Similarly, since products of the CAM are not retrieved in administrative databases, our study was not able to detect their use among MG patients. Regarding the analysis of determinants, it was not possible to create an overall dataset and perform a pooled analysis due to recently introduced restrictions in the use of regional administrative healthcare data by data protection officers. We considered performing a meta-analysis of the regional results, but numbers were too small for the single outcomes. Finally, our study did not evaluate safety issues derived from the use of pyridostigmine in presence of contraindications or the use of potentially contraindicated drugs in MG patients, such as the occurrence of ADRs or myasthenic crisis. In general, population-based studies provide valuable insights into the management and outcomes of MG patients. However, they also have inherent limitations, including confounding factors. Therefore, caution should be exercised when interpreting and generalizing results of cohort studies, which should be complemented by other study types, such as systematic reviews and meta-analyses.

On the other hand, to the best of our knowledge, no prior publications on this topic exist in Italy, and only a limited number of international studies are available, the majority of which focused only on drug use patterns in MG rather than on the use of prescription drugs in presence of contraindications. The main strength of our study lies in its population-based approach and the inclusion of data regarding the resident population, covering significant demographic and clinical characteristics of MG patients. Furthermore, the identified cohorts are not biased or selectively chosen, and the analysis comprises a substantial sample of individuals affected by MG, despite it being a rare disease. In fact, this is a multicentre study offering evidence from various regions in Italy, which collectively represent approximately 18% of the country’s residents. Thus, our analysis allows for generalization of the results to the entire population of the country. Lastly, although information on individual dosages is unavailable in our databases, the medications under investigation are all reimbursed by the healthcare system and have thus undergone thorough evaluation.

## 5 Conclusion

Our analysis examined the drug use patterns of pyridostigmine and potentially inappropriate medications in individuals with MG. We found that over 15% of incident pyridostigmine users had contraindications, and during the first year of follow-up, over 60% received potentially contraindicated drugs. Factors such as increasing age, specific complications or comorbidities, polytherapy, and non-pharmacological therapy were associated with receiving pyridostigmine with contraindications. Determinants for using potentially contraindicated drugs in MG included increasing age, specific complications or comorbidities, specific pharmacological therapy, and polytherapy. Future studies will assess safety issues tied to pyridostigmine use with contraindications or concurrent administration of inappropriate medications in individuals with MG, including therapy discontinuation, ineffectiveness, and adverse drug reactions. This information aims to raise healthcare professionals’ awareness of the appropriate use of medications, over-the-counter products, and complementary and alternative medicines in this vulnerable population.

## Data availability statement

The raw data supporting the conclusions of this article will be made available by the authors, without undue reservation.

## Author contributions

GC: Conceptualization, Methodology, Writing – original draft, Writing – review & editing. MF: Data curation, Formal analysis, Writing – original draft, Writing – review & editing. OP: Data curation, Formal analysis, Writing – review & editing. PB: Supervision, Visualization, Writing – review & editing. FS: Supervision, Visualization, Writing – review & editing. MT: Supervision, Validation, Writing – review & editing. AA: Supervision, Validation, Writing – review & editing. AV: Supervision, Validation, Writing – review & editing. NL: Conceptualization, Methodology, Writing – original draft, Writing – review & editing. UK: Conceptualization, Funding acquisition, Methodology, Project administration, Validation, Writing – original draft, Writing – review & editing.

## CAESAR Study group

Antonio Addis, Antonio Ancidoni, Ilaria Bacigalupo, Anna Maria Bargagli, Valeria Belleudi, Roberto Bonaiuti, Paola Brunori, Giampaolo Bucaneve, Teresa Anna Cantisani, Silvia Cascini, Maria Grazia Celani, Livia Convertino, Giada Crescioli, Marina Davoli, Marco Finocchietti, Rosa Gini, Giulia Hyeraci, Ursula Kirchmayer, Niccolò Lombardi, Olga Paoletti, Rosalba Elisabetta Rocchi, Mariangela Rossi, Francesco Sciancalepore, Marco Tuccori, Nicola Vanacore, and Alfredo Vannacci.
