# Supplementary material for: Potentially inappropriate drug use in myasthenia gravis: a real-world population-based cohort study in Italy
Source: Front Neurol. 2023 Dec 21;14:1293626. doi: 10.3389/fneur.2023.1293626 (PMC10764541; doi:10.3389/fneur.2023.1293626)
Supplement: Supplementary file 1 [file Table_1.DOCX]

Supplementary Material

**Supplementary Table 1.** Contraindications for Py use.

|  | **Pyridostigmine incident users** | |
| --- | --- | --- |
|  | **591** | |
|  | **n** | **%** |
| **Contraindications** | | |
| Mechanical gastrointestinal obstruction | 1 | 0.2% |
| Mechanical urinary obstruction | 0 | 0.0% |
| Obstructive respiratory diseases | 20 | 3.4% |
| Cardiovascular diseases | 79 | 13.4% |
| Care involving breathing exercises | 0 | 0.0% |

**Supplementary Table 2.** Potentially contraindicated drugs in MG.

|  | **MG subjects** | |
| --- | --- | --- |
|  | **2369** | |
|  | **n** | **%** |
| **Drugs with contraindications** | | |
| **Drugs for the cardiovascular system** | **902** | **38.1%** |
| Beta blocking agents (ATC code: C07) | 430 | 18.2% |
| Calcium channel blockers (ATC code: C08) | 364 | 15.4% |
| Procainamide (ATC code: C01BA02) | 0 | 0.0% |
| Disopyramide (ATC code: C01BA03) | 0 | 0.0% |
| Statins (ATC code: C10AA) | 466 | 19.7% |
| **Drugs for the nervous system** | **704** | **29.7%** |
| Antiepileptics (ATC code: N03) | 283 | 11.9% |
| Antipsychotics (ATC code: N05A) | 90 | 3.8% |
| Antidepressants (ATC code: N06A) | 528 | 22.3% |
| **Immunosuppressants (ATC code: L04A)** | **507** | **21.4%** |

**Supplementary Table 3.** ICD-9-CM codes.

| **Disorder** | **ICD-9-CM diagnosis code** |
| --- | --- |
| Myasthenia gravis | 358.0 |
| Mechanical gastrointestinal obstruction | 560.0, 560.9 |
| Mechanical urinary obstruction | 593.4 |
| Obstructive respiratory diseases | 490, 491, 492, 493, 494, 496 |
| Cardiovascular diseases | 410, 411, 412, 413, 414, 427, 428, 429 |
| Care involving breathing exercises | V570 |
| Acute respiratory failure | 518.81 |
| Myasthenia gravis with (acute) exacerbation | 358.01 |
| Benign neoplasm of thymus | 212.6 |
| Malignant neoplasm of thymus | 164.0 |
| Thyrotoxicosis | 242.9, 242.0 |
| Chronic lymphocytic thyroiditis | 245.2 |
| Systemic lupus erythematosus | 710.0 |
| Rheumatoid arthritis | 714.0 |
| Pernicious anemia | 281.0 |
| Other chronic hepatitis | 571.49 |
| Psoriasis and similar disorders | 696 |
| Diabetes with neurological manifestations | 250.6 |
| Malignant essential hypertension | 401.0 |
| Diabetes mellitus without mention of complication | 250.0 |
| Toxic cataract | 366.45 |
| Other osteoporosis | 733.09 |
| Fractures | 800-829 |
| Cushing's syndrome | 255.0 |
| Poisoning with parasympathomimetics [cholinergics] | 971.0 |
| Obstructive chronic bronchitis | 491.2 |
| Other and unspecified noninfectious gastroenteritis and colitis | 558 |
| Other specified cardiac dysrhythmias | 427.8 |
| Other and unspecified hyperlipidemia | 272.4 |
| Overweight, obesity and other hyperalimentation | 278 |
| Cramp of limb | 729.82 |
| Autoimmune diseases | 135, 136.1, 250.11, 250.13, 250.31, 250.33, 250.41, 250.43, 250.51, 250.53, 250.71, 250.73, 250.81, 250.83, 250.91, 250.93, 266.2, 281.1, 283, 287, 340, 341, 446, 447.6, 555, 556, 694.4, 695.4, 704, 709.01, 710.1, 710.2, 710.4, 720 |
| Liver diseases | 070.22, 070.23, 070.32, 070.33, 070.44, 070.54, 070.6, 070.9, 456.0, 456.1, 456.2, 570, 571, 572.2, 572.3, 572.4, 572.5, 572.6, 572.7, 572.8, 573.3, 573.4, 573.8, 573.9, V427 |
| Renal diseases | 274.10, 572.4, 580, 581, 582, 583, 584, 585, 586, 587, 588, 593, 753.0, 753.3, 791.2, 791.3, 866.00, 866.01, 866.1, V420, V451, V56 |
| Neoplasms | 140-239 |
| Mental disorders | 290-319 |
| Inflammatory diseases of the central nervous system | 320-326 |
| Diseases of the circulatory system | 390-459 |
| Diseases of the respiratory system | 460-519 |
| **Procedure** | **ICD-9-CM procedure code** |
| Thymectomy | 07.8 |
| Non-invasive mechanical ventilation | 93.90 |
| Invasive mechanical ventilation | 96.7 |
| Plasmapheresis | 99.71 |
|  |  |
